# Parent Report and Actigraphically Defined Sleep in Children with and without Developmental Coordination Disorder; Links with Fatigue and Sleepiness

**DOI:** 10.3389/fped.2016.00081

**Published:** 2016-08-04

**Authors:** Luci Wiggs, Masako Sparrowhawk, Anna L. Barnett

**Affiliations:** ^1^Department of Psychology, Social Work and Public Health, Faculty of Health and Life Sciences, Oxford Brookes University, Oxford, UK

**Keywords:** developmental coordination disorder, sleep, actigraphy, fatigue, sleepiness, pre-sleep arousal, restless legs syndrome, adolescents

## Abstract

**Background:**

Impaired sleep is associated with negative effects on quality of life and daytime functioning. Higher rates of sleep disturbance are reported in children with various developmental disorders. However, little is known about sleep in children with developmental coordination disorder (DCD), a condition characterized by everyday movement difficulties. Previously, in a preliminary study, we found higher rates of parent-reported sleep disturbance in children with DCD compared to controls.

**Aims:**

To examine sleep in DCD using objective measures and to examine links with daytime fatigue and sleepiness.

**Methods:**

Two groups (primary and secondary school-aged) of 15 children with DCD, plus matched controls, participated. Parent-reported child sleep was assessed using the Children’s Sleep Habits Questionnaire and actigraphy provided an objective measure of sleep–wake patterns over 1 week (including weekdays and weekend). Pediatric restless legs syndrome (RLS) semi-structured diagnostic interview was conducted with each child and parent to capture symptoms of RLS. Aspects of self-rated child functioning were assessed with questionnaires (Pre-sleep Arousal Scale, Pediatric Daytime Sleepiness Scale, PedsQL Multidimensional Fatigue Scale) and mothers’ reported thoughts about child sleep with the Maternal Cognitions about Infant Sleep Questionnaire.

**Results:**

The DCD groups had greater parent-reported sleep disturbance. Actigraphy results suggested that for secondary aged children with DCD their sleep quality was impaired and there were differences in the timing of sleep compared to controls (including some differences in the variation between weekday and weekend sleep times). The actigraphy of the primary age group with DCD was unremarkable compared to controls. No child in the study met the criteria for RLS. Exploratory analyses suggested that daytime fatigue, aspects of pre-sleep arousal, and daytime sleepiness were reported as greater in the DCD groups and were particularly related to objective sleep parameters in the DCD groups. Maternal thoughts about sleep did not differ between the DCD and control groups.

**Conclusion:**

The nature and underlying cause of sleep disturbance and how it might be linked with aspects of daytime functioning in adolescents with DCD requires further research. Meanwhile, clinical awareness of the risk of atypical sleep patterns/sleep problems in DCD is important to ensure early identification and implementation of appropriate support.

## Introduction

Developmental coordination disorder (DCD) is recognized by the American Psychiatric Association as a significant difficulty in the learning of motor skill in the absence of sensory, intellectual or neurological impairment (1). The motor difficulties are apparent from early in childhood and often persist into adolescence and adulthood (2). These have an impact on everyday self-care activities, such as eating and dressing as well as leisure activities, education, and employment. Varying prevalence rates for DCD have been reported, due to the use of different assessment methods and cut-off points (1, 3). However, a population study in the UK strictly based on criteria from the DSM (4) reported a prevalence of almost 2% in primary school children (5).

National and international consensus statements and guidelines have led to the use of more uniform and rigorous methods of diagnosis and assessment in DCD research (3, 6). An abundance of studies have investigated the nature of the motor control and coordination difficulties (7) to gain a better understanding of the core feature of the condition. Although the motor difficulties of children with DCD are troubling in themselves, there is a range of other difficulties very commonly associated with the condition that may cause even greater concern for the child’s general progress and their physical and psychological well-being. It is now reasonably well established that DCD is associated with lower levels of physical activity and an increased incidence of children who are over-weight or obese (8, 9). This is associated with lower levels of cardio-respiratory fitness and concerns for long-term physical health (10). A range of psychological, social, and emotional problems are also frequently reported in individuals with DCD. These include low self-esteem (11), difficulties with peer relations (12), anxiety (13), and depression (14). Such problems have been well documented in the literature and can persist through adolescence (2). Indeed, more severe psychiatric problems have been reported in studies on adults with DCD (15).

Although there is substantial descriptive information available, it is only recently that explanatory frameworks have been suggested to help understand the nature of the relationships between the range of motor, physical, and psycho-social difficulties reported in DCD. Cairney and colleagues (10, 16) adopt Pearlin’s stress process model (17, 18) to illustrate possible direct and indirect links between exposure to the stress linked with DCD and emerging health and psychological problems. However, one important factor that has not yet been explored but may be very relevant to understanding the range of difficulties associated with DCD is the role of sleep and sleep disturbance.

Sleep disturbance has been found to be common in groups of children with other neurodevelopmental disorders and psychiatric disturbances, such as anxiety and depression (19). Importantly, impaired sleep in various groups of young people has been found to be associated with impairments in cognitive functioning and psychological well-being [e.g., Ref. (20–22)] as well as physical health [e.g., Ref. (23)]. Furthermore, some of these relationships are likely to be bi-directional [e.g., sleep and depression (24)], with some interplay between sleep, physical health, and psychological factors (25, 26). Since children’s sleep has also been found to contribute to aspects of motor learning (27), including observational learning (28), this is a further reason to examine the amount and quality of sleep in children with motor difficulties, such as DCD.

It is perhaps surprising that sleep has received so little attention in DCD, given both its co-occurrence with other disorders known to be associated with sleep disturbance (19) and concerns about the range of physical and psychological problems associated with DCD. One EEG study has reported epileptiform activity during sleep in DCD (29) but the only investigation of the quantity and quality of sleep in children with DCD comes from our previous exploratory questionnaire study of 16 primary school-aged boys with DCD (30). We found that parent-reported difficulties with sleep were more prevalent for boys with DCD compared to the control group. Subscale scores indicated particular problems with bedtime resistance, parasomnias and daytime sleepiness. These preliminary results suggest that sleep patterns of children with DCD may be of clinical relevance and are worthy of further investigation.

The primary aim of the current study was, therefore, to investigate sleep in children with DCD, using actigraphy as an objective measure of sleep timing and pattern. Given the sleep anomalies suggested by the results of our earlier study and also that children with DCD have poorer psychological well-being emphasis was placed on trying to better understand the sleep of children with DCD in the hope that the results would inform future studies addressing the links between these areas. Additional aspects of sleep investigated included features associated with children’s sleep onset difficulties [including pre-sleep arousal, symptoms of restless legs syndrome (RLS), and parental thoughts about child sleep] and also a more careful description of any daytime “sleepiness” to try and distinguish actual sleepiness (i.e., propensity to fall asleep) from fatigue (i.e., lack of energy and motivation), arising as a result of physical or mental exhaustion. Because developmental changes in sleep have been documented during adolescence for a variety of biological and social factors (31); in this study, we examined sleep separately in younger and older school age groups. Relationships between overnight sleep and daytime sleepiness/fatigue were also investigated in an exploratory manner.

## Materials and Methods

### Participants and Selection Measures

Sixty children took part in the study; half were in primary school (7–11 years) and half in secondary school (11–16 years) in the UK. In addition, one child with DCD was recruited into the study but could not comply with wearing an actigraph so was excluded and one control child was excluded because, although he had no formal diagnosis, he was reported by teachers to have severe emotional and behavioral problems.

In each age group, 15 children met the diagnostic criteria for DCD. For each child with DCD, an age (within 6 months) and gender match without motor difficulties was selected to be part of the control group. Children with epilepsy, currently taking prescribed medication for sleep disturbances or who consistently shared a bed with anyone else were excluded from the study. Details of each group can be found in Table 1.

**Table 1:** **Characteristics of primary and secondary school age children in the DCD and control groups**.

	DCD group		Control group	
	Primary	Secondary	Primary	Secondary
Number	15	15	15	15
Males:females	15:0	10:5	15:0	10:5
Age in months mean (SD)	112.9 (13.9)	170.2 (18.4)	111.8 (12.7)	170.4 (19.45)
Age in years:months (Min–Max)	9:5 (7:5–11:3)	14:2 (11:11–16:9)	9:4 (7:9–10:11)	14:2 (12:4–17:0)
MABC-2 Test mean standard score (SD)	4.27 (1.16)	5.27 (1.22)	10.93 (2.55)	12.67 (2.64)
MABC-2 mean percentile	3.63	7.13	58.73	73.93
BPVS-3 mean (SD)	102.40 (11.08)	106.67 (11.31)	101.53 (15.19)	105.27 (12.86)

*MABC-2, Movement Assessment Battery for Children second edition*.

*BPVS 3, British Picture Vocabulary Scale third edition*.

All children with DCD were recruited from a database of children with DCD who had previously taken part in research at the University. They were originally recruited from three sources: (1) our research group website, (2) local schools, and (3) a local support group for individuals with movement difficulties. All participants with DCD were assessed and selected in line with the DSM-5 criteria for DCD (1) and recent European guidelines (3). Different assessments were used by a chartered psychologist with extensive expertise in DCD to ensure that each of the four diagnostic criteria was met. For criterion A, we carried out the test component of the Movement Assessment Battery for Children second edition [MABC-2; (32)], which has UK norms for individuals up to 16 years of age. The group of participants with DCD all scored at or below the 16th percentile on the MABC-2. The Movement ABC-2 Checklist describes the level of performance on a range of everyday motor tasks at home and school. This was used, together with a telephone interview with the child’s parent to determine that the motor impairment significantly impacted on activities of daily living (criterion B) and that the onset of these difficulties was during early childhood (criterion C). The telephone interview with parents also ensured that the difficulties were not due to a known neurological impairment or intellectual disability (criterion D).

Children in the control group were recruited from three sources: (1) a database of children who have taken part in previous studies at the University, (2) personal contacts of researchers, and (3) local schools. The typically developing (TD) control children were age (to within 6 months) and gender matched to each participant with DCD. A telephone interview and the Movement ABC-2 Test and Checklist were used to confirm that no movement difficulties were present. All children scored above the 16th percentile on both the Movement ABC-2 Test and Checklist (32), indicating typical levels of motor competence for their age. Children with known developmental disorders or severe medical/psychiatric conditions were excluded.

In addition, for both groups, the British Picture Vocabulary Scale – 3rd Edition [BPVS-3; (33)] was used to obtain a measure of receptive vocabulary, which correlates highly with Verbal IQ (34). All children had to obtain a standard score of at least 80, indicating verbal ability at the 9th percentile or above. The Strength and Difficulties Questionnaire [SDQ; (35)] was used to record other behavioral difficulties noted by the parent. This is a brief behavioral screening tool for children aged 4–16 years, assessing behavior problems relating to conduct, emotional control, peer relationships, hyperactivity/inattention, and prosocial behavior. A Total Difficulties Score indicates behavior in the “normal,” “borderline,” or “abnormal” range.

### Sleep Measures

#### Actigraphy

The actigraph (Micro Mini Motionlogger, Ambulatory Monitoring Inc.), an accelerometer, was worn on the non-dominant wrist for at least seven nights from just before the child went to bed until shortly after they woke in the morning. Movement was monitored continuously and stored within the unit. Subsequent analysis of frequency and pattern of movement by means of validated algorithms permits detection of basic sleep–wake patterns (36). Movements were scored in 1-min epochs; all epochs that are scored above a pre-set threshold (Sadeh’s algorithm applied by the software) are scored as “wake” and those that are below this threshold are scored as “sleep.”

Actigraphy data were interpreted in the light of a sleep diary, which recorded children’s bed time, light-off time (or “snuggling down to sleep” time), time fallen asleep and wake up time and duration of wakes during the night. Diaries were completed by parents or self-completed by older children.

The start of sleep was determined from the actigraph as the first epoch in the first 10-min interval, after bedtime (recorded in the diary) where there is no more than 1 epoch that is above the threshold (automatically calculated by the software) for wake. Any sleep interruptions were determined by the actigraph by searching for 10-min intervals in which activity in more than 1 epoch is scored as “wake.” Sleep offset (i.e., final waking time) is determined as the last epoch in the last 10-min period prior to “get up time” (recorded in the diary) in which there was no more than 1 epoch that was scored as “wake.” The last minute of this 10-min period provided the sleep offset time.

Scores were averaged over the recording nights for each child [excluding any nights which were atypical (e.g., child was ill)]. The variables generated were: *Bedtime* (time to settle down to try and initiate sleep; primarily from diary, validated by actigraphy), *sleep onset* and *wake up* times, *sleep latency* (minutes between snuggling down to sleep and sleep onset), *sleep duration* in minutes (total sleep minutes between onset and offset minus minutes of wake), *wake minutes* (minutes of wake between bedtime and sleep offset), *wake after sleep onset* (WASO) (minutes of wake between sleep onset and offset), *wake episodes* (number of contiguous wakes after sleep onset), *sleep efficiency* (percentage of time in bed spent asleep), *fragmentation index* (number of wakes divided by total sleep minutes × 100), *activity mean* (amount of movement during sleep), and *standard deviation of activity mean* [within-subject night-to-night variability for this measure was analyzed because previous work has suggested greater sleep instability in some groups of children, e.g., ADHD (37)].

#### Children’s Sleep Habits Questionnaire

This is a standardized, 52-item tool used to screen for sleep problems in school-age children (38). Parents are asked to recall events over the past typical week. Responses require parents to either write in their child’s bed, sleep, and wake times or to indicate the frequency of occurrence of a number of sleep-related behaviors on a three-point scale ranging from “usually”/5–7 times a week to “sometimes”/2–4 times a week and “rarely”/0–1 times a week (scored 3, 2, and 1, respectively). The scoring of some items is reversed so that a higher score is indicative of more problematic sleep.

In addition to information about sleep timing, the questionnaire gives both an overall sleep disturbance score (ranging from 33 to 99) and eight subscale scores: bedtime resistance (six items), sleep onset delay (one item), sleep duration (three items), sleep anxiety (four items), night wakings (three items), parasomnias (seven items), sleep-disordered breathing (three items), and daytime sleepiness (eight items), reflecting key areas of clinical concern. Owens and colleagues’ (38) analysis of the total CSHQ scores of a community sample of 469 school-aged children and 154 patients diagnosed with sleep disorders in a pediatric sleep clinic suggested that a cut-off total sleep disturbance score of 41 gives the best diagnostic confidence of identifying children with sleep disturbance of clinical significance, with a sensitivity of 0.80 and specificity of 0.72.

#### Pediatric Restless Legs Syndrome Semi-Structured Diagnostic Interview

Restless legs syndrome is characterized by an urge to move the legs, frequently associated with other sensations (39). These sensations are worse when at rest, relieved by movement and most severe at night. This 24-item interview incorporates information from children and parents allowing the categorization of children and adolescents meeting the RLS consensus criteria (39, 40). To ensure that children 12 years old and younger are not simply agreeing to a description of RLS symptoms, this age group must describe the sensory component in their own words. For children and adolescents who do not meet their age specific criteria set, additional research criteria of “possible RLS” and “probable RLS” may also be applied. Areas of enquiry and wording of questions were derived from empirical data gathered by Picchietti et al. (41). An additional parent question asking if the child had had polysomnography (PSG) documenting a periodic limb movement (PLM) index of 5 or more per hour of sleep was added, with further details obtained if appropriate.

#### Pediatric Daytime Sleepiness Scale

This is an 8-item self-report instrument designed for use with children of middle-school age (11–15 years), which has been widely used with a wider age range, including from age 8 to 18 (42). Items assess the frequency of sleepiness related behaviors (e.g., falling asleep or getting drowsy during class periods; thinking you need more sleep) using a scale (0 = never, 4 = always), with higher scores indicating increased sleepiness. Mean score values in the original study were 15.3 ± 6.2. The PDSS has shown both acceptable internal consistency as well as expected associations with outcomes linked to sleepiness (e.g., decreased sleep time, poor grades, negative moods) (43).

#### PedsQL Multidimensional Fatigue Scale

This self-report instrument measures three aspects of fatigue: General Fatigue (e.g., I feel tired, I feel physically weak.), Sleep/Rest Fatigue (e.g., I feel tired when I wake up in the morning, I rest a lot), and Cognitive Fatigue (e.g., It’s hard for me to keep attention on things, It’s hard for me to remember what people tell me) (44). It also gives a Total Fatigue score, which is an average of the three subscales. Each subscale has six items rated on a five-point scale from 0 (never) to 4 (almost always). Item scores are transformed on a scale from 0 to 100 (0 = 100, 1 = 75, 2 = 50, 3 = 25, 4 = 0) and a mean obtained for each subscale with lower scores indicating greater fatigue. High internal consistency and concurrent validity have been reported (45).

#### Pre-Sleep Arousal Scale

This measures somatic arousal (e.g., “cold feeling in your hands, feet, or your body in general”) and cognitive arousal (e.g., “worry about falling asleep”) (46). Sixteen items (eight for each subscale) are rated on a five-point scale (1 = not at all to 5 = extremely) with scores ranging from 8 to 40 for each subscale, with higher scores indicating increased arousal in the pre-sleep period. As this measure was originally designed for use with adults, an adaptation for children was used (47). Adaptations were word substitutions and the inclusion of explanations for some terms and concepts. The child version of the scale has been shown to have reasonable internal consistency (total scale α = 0.85; each subscale α = 0.75).

#### Maternal Cognitions about Infant Sleep Questionnaire

This assesses parents’ agreement (rated on a five-point scale of “Strongly agree” to “Strongly disagree”) with 20 statements concerning their attitudes and cognitions relating to their child’s sleep (48). Five subscales are derived as follows: (i) problems with setting limits (scores can range from 0 to 25); (ii) anger directed at the child (0–25); (iii) doubts concerning maternal competence (0–25); (iv) concerns about the child’s safety (0–10), and (v) issues related to night-feeding (0–15). Some items are reverse scored and higher scores indicate more problematic cognitions. The questionnaire was designed for use with parents of TD infants but was modified for use with older children by removing the three items concerning night-feeding. It has been found to be sensitive in detecting different profiles of cognitions in parents of young children with and without sleep disorders (48).

### Procedure

Ethical approval for the study was given by the University Research Ethics Committee at Oxford Brookes University. Where parent and child consented to take part, the child’s family doctor was informed. If there was no objection from the family doctor, then a telephone interview was conducted by the second author with the parent to confirm the child’s suitability to participate. Subsequently, a home visit was arranged to undertake the testing and administer questionnaires with the child and parent. For the following seven nights, the parents were asked to keep a sleep diary and the child to wear an actigraph. All recording was undertaken during school term time, avoiding school holidays.

### Data Analysis

Differences between the DCD and control groups for all variables were examined separately for the primary aged and secondary aged children. Actigraph analyses were based on a mean number of 6.87 nights (SD = 0.51) in the control group and 6.53 nights (SD = 0.86) in the DCD group. Since patterns of sleep during the week and weekend may differ in children (49, 50) the weekday and weekend data (Friday and Saturday nights) were examined separately (data presented in Tables 2 and 3). For analysis of all actigraphy-dependent variables in both the primary and secondary age groups, two-way 2 (group: DCD or control) × 2 (time: weekday or weekend) mixed ANOVAs were used. Wilks’ Lambda F statistics are presented in Tables 2 and 3 along with partial eta squared effect sizes. Of note, the secondary aged weekend *bedtime* scores violated the assumption of equality of variances and so these results need to be treated with caution. To limit type 1 errors as a result of multiple comparisons, false discovery rate (FDR) control was enforced (51). A 0.05 FDR level was set, i.e., the null hypothesis would be true of a maximum of 5% of significant results. In this procedure, the rank of each *p*-value is divided by the number of tests (*n* = 36) and multiplied by 0.05; only where the obtained *p*-value is less than the derived value is the result considered statistically significant [see (52) for a discussion of this method].

**Table 2:** **Actigraphically derived sleep variable mean scores (and standard deviations) for primary aged children during weekdays and weekends**.

	DCD primary group		Control primary group		ANOVA statistics	*p*-value	Effect size $\eta_{p}^{2}$
	Weekday (*n* = 15)	Weekend (*n* = 15)	Weekday (*n* = 14)	Weekend (*n* = 14)			
Bedtime (hours:minutes^a^)	20:46 (40.2)	21:25 (40.8)	20:49 (25.8)	21:26 (34.8)	Time *F*(1, 27) = 31.317	**<0.0005**	0.537
					Group *F*(1, 27) = 0.074	0.788	0.003
					Time × group *F*(1, 27) = 0.068	0.796	0.003
Sleep start time (hours:minutes^a^)	21:16 (40.8)	22:03 (43.2)	21:25 (36.6)	21:59 (40.2)	Time *F*(1, 27) = 42.354	**<0.0005**	0.611
					Group *F*(1, 27) = 0.025	0.875	0.001
					Time × group *F*(1, 27) = 1.102	0.303	0.039
Wake up time (hours:minutes^a^)	6:46 (39.0)	6:35 (52.2)	7:01 (24.6)	7:20 (46.2)	Time *F*(1, 27) = 0.490	0.490	0.018
					Group *F*(1, 27) = 4.325	*0.047*	0.138
					Time × group *F*(1, 27) = 5.724	*0.024*	0.175
Latency (minutes^a^)	31.06 (17.10)	30.13 (19.95)	40.57 (31.18)	32.54 (21.30)	Time *F*(1, 27) = 1.009	0.324	0.036
					Group *F*(1, 27) = 0.462	0.503	0.017
					Time × group *F*(1, 27) = 0.574	0.455	0.021
Sleep duration (minutes^a^)	489.08 (45.99)	440.87 (65.64)	504.96 (41.78)	492.50 (39.11)	Time *F*(1, 27) = 11.080	**0.003**	0.291
					Group *F*(1, 27) = 4.434	*0.045*	0.141
					Time × group *F*(1, 27) = 3.847	0.060	0.125
Wake minutes^a^	134.17 (60.10)	138.50 (58.31)	124.64 (51.09)	132.07 (39.94)	Time *F*(1, 27) = 0.440	0.513	0.016
					Group *F*(1, 27) = 0.182	0.673	0.007
					Time × group *F*(1, 27) = 0.023	0.882	0.001
Wake after sleep onset (minutes^a^)	81.28 (56.22)	71.93 (45.69)	62.47 (31.39)	71.54 (38.91)	Time *F*(1, 27) = 0.040	0.843	0.001
					Group *F*(1, 27) = 0.311	0.582	0.011
					Time × group *F*(1, 27) = 1.823	0.188	0.063
Wake episodes	5.81 (1.65)	5.60 (1.80)	5.54 (1.17)	5.64 (1.26)	Time *F*(1, 27) = 0.104	0.750	0.004
					Group *F*(1, 27) = 0.023	0.881	0.001
					Time × group *F*(1, 27) = 0.157	0.695	0.006
Sleep efficiency (%)	78.32 (7.38)	76.32 (10.27)	80.47 (7.55)	78.91 (5.92)	Time *F*(1, 27) = 1.479	0.235	0.052
					Group *F*(1, 27) = 0.767	0.389	0.028
					Time × group *F*(1, 27) = 0.041	0.841	0.002
Fragmentation index	1.23 (0.40)	1.30 (0.44)	1.12 (0.30)	1.17 (0.31)	Time *F*(1, 27) = 0.561	0.460	0.020
					Group *F*(1, 27) = 0.794	0.381	0.029
					Time × group *F*(1, 27) = 0.136	0.716	0.005
Activity mean	18.34 (7.17)	17.30 (6.42)	15.86 (4.87)	16.20 (4.57)	Time *F*(1, 27) = 0.306	0.584	0.011
					Group *F*(1, 27) = 0.665	0.422	0.024
					Time × group *F*(1, 27) = 0.269	0.608	0.010
Activity SD	37.60 (11.02)	35.76 (9.15)	34.89 (7.73)	35.94 (7.67)	Time *F*(1, 27) = 0.187	0.669	0.007
					Group *F*(1, 27) = 0.102	0.752	0.004
					Time × group *F*(1, 27) = 0.427	0.519	0.016

*ANOVA results [for time (weekend vs. weekday), group (DCD vs. controls) and interaction effects], *p*-values and effect sizes (partial Eta squared $\eta_{p}^{2}$)*.

*^a^Fractions of a minute are decimalized in the table; time = weekday vs. weekend; Group = DCD vs. control; p-values <0.05 which remained significant at the 0.05 false discovery rate are marked in bold for ease of reading. Those in italics failed to reach significance following this control procedure*.

**Table 3:** **Actigraphically derived sleep variable mean scores (and SDs) for secondary aged children during weekdays and weekends**.

	DCD secondary group		Control secondary group		ANOVA statistics	*p*-value	Effect size $\eta_{p}^{2}$
	Weekday (*n* = 15)	Weekend (*n* = 15)	Weekday (*n* = 14)	Weekend (*n* = 14)			
Bedtime^a^ (hours:minutes^b^)	21:48 (43.8)	22:14 (40.2)	22:07 (45.0)	22:58 (69.0)	Time *F*(1, 27) = 18.695	**<0.0005**	0.409
					Group *F*(1, 27) = 3.757	0.063	0.122
					Time × group *F*(1, 27) = 1.762	0.195	0.061
Sleep start time (hours:minutes^b^)	22:36 (42.0)	22:45 (46.8)	22:38 (61.2)	23:38 (67.2)	Time *F*(1, 27) = 15.899	**<0.0005**	0.371
					Group *F*(1, 27) = 2.254	0.145	0.077
					Time × group *F*(1, 27) = 8.408	**0.007**	0.237
Wake up time (hours:minutes^b^)	6:46 (28.8)	7:56 (51.6)	6:53 (23.4)	8:58 (64.8)	Time *F*(1, 27) = 91.351	**<0.0005**	0.772
					Group *F*(1, 27) = 6.681	**0.015**	0.198
					Time × group *F*(1, 27) = 7.882	**0.009**	0.226
Latency (minutes^b^)	46.32 (25.92)	36.57 (22.97)	30.72 (21.91)	38.75 (30.03)	Time *F*(1, 27) = 0.025	0.876	0.001
					Group *F*(1, 27) = 0.696	0.411	0.025
					Time × group *F*(1, 27) = 3.435	0.075	0.113
Sleep duration (minutes^b^)	440.46 (55.07)	462.03 (68.50)	456.15 (60.83)	502.21 (95.09)	Time *F*(1, 27) = 6.962	**0.014**	0.205
					Group *F*(1, 27) = 1.394	0.248	0.049
					Time × group *F*(1, 27) = 0.960	0.336	0.034
Wake minutes^b^	112.28 (48.20)	145.6 (46.31)	89.53 (47.93)	119.32 (76.65)	Time *F*(1, 27) = 19.511	**<0.0005**	0.419
					Group *F*(1, 27) = 1.839	0.186	0.064
					Time × group *F*(1, 27) < 0.000	0.996	0.000
Wake After Sleep Onset (minutes^b^)	50.78 (33.77)	80.61 (40.47)	41.86 (30.71)	55.43 (58.39)	Time *F*(1, 27) = 13.564	**0.001**	0.334
					Group *F*(1, 27) = 1.620	0.214	0.057
					Time × group *F*(1, 27) = 1.253	0.273	0.044
Wake episodes	5.39 (1.59)	6.23 (1.44)	4.39 (1.71)	4.07 (1.88)	Time *F*(1, 27) = 1.041	0.317	0.037
					Group *F*(1, 27) = 10.143	**0.004**	0.273
					Time × group *F*(1, 27) = 2.421	0.131	0.082
Sleep efficiency (%)	79.74 (8.60)	75.86 (7.94)	83.64 (8.83)	81.11 (12.09)	Time *F*(1, 27) = 9.808	**0.004**	0.266
					Group *F*(1, 27) = 2.053	0.163	0.071
					Time × group *F*(1, 27) = 0.164	0.689	0.006
Fragmentation index	1.29 (0.48)	1.41 (0.51)	1.02 (0.47)	0.90 (0.54)	Time *F*(1, 27) = 0.023	0.881	0.001
					Group *F*(1, 27) = 5.814	*0.023*	0.177
					Time × group *F*(1, 27) = 1.513	0.229	0.053
Activity mean	16.02 (5.86)	20.04 (8.03)	14.58 (5.83)	16.23 (9.92)	Time *F*(1, 27) = 8.086	**0.008**	0.230
					Group *F*(1, 27) = 1.080	0.308	0.038
					Time × group *F*(1, 27) = 1.090	0.306	0.039
Activity SD	33.97 (10.81)	39.76 (12.87)	31.25 (9.02)	32.39 (14.13)	Time *F*(1, 27) = 5.635	*0.025*	0.173
					Group *F*(1, 27) = 1.543	0.225	0.054
					Time × group *F*(1, 27) = 2.103	0.159	0.072

*ANOVA results [for time (weekend vs. weekday), group (DCD vs. controls) and interaction effects], *p*-values and effect sizes (Partial Eta Squared $\eta_{p}^{2}$)*.

*^a^Unequal variances for weekend bedtime so ANOVA statistics to be treated with caution*.

*^b^Fractions of a minute are decimalized in the table; Time = weekday vs. weekend; Group = DCD vs. control; p-values <0.05 which remained significant at the 0.05 false discovery rate are marked in bold for ease of reading. Those in italics failed to reach significance following this control procedure*.

For all other, non-actigraphy variables where group differences were examined in an exploratory manner, *t*-tests or non-parametric Mann–Whitney *U* tests were employed as appropriate. Relationships between the measures were examined using Pearson or Spearman correlations as appropriate. The effect of severity of motor impairment in the DCD group on the sleep measures was also examined by using *t*-tests to compare scores of the children scoring above and below the 5th percentile on the MABC-2 (the recommended cut-off to indicate the most severe degree of impairment on this test). The significance level was set at *p* ≤ 0.05 for all tests but the effect of multiple comparisons needs to be considered when interpreting the results.

## Results

Eight children in the DCD group had MABC-2 scores at or below the 16th percentile and 22 at or below the 5th. Within the DCD group five primary and four secondary-aged children were reported by their parent to have an additional diagnosis of one or more of the following conditions: dyslexia (five children), attention deficit hyperactivity disorder (ADHD; two children), and autistic spectrum disorder (ASD; four children). One of the children with ADHD took stimulant medication (methylphenidate). Planned analyses were run both including and excluding the children with ADHD and ASD. Since excluding the children did not change the significance of any results all children were included in the presented analyses below. No control children, but 11 children with DCD (3 primary and 8 secondary aged), had SDQ total scores in the “abnormal” range. Difficulties were noted across all areas assessed, although hyperactivity/inattention and peer problems were most commonly reported. There were no significant differences between the DCD and control groups in their BPVS scores (*t* = 55.414; *p* = 0.730). Details of each group can be found in Table 1.

### Actigraphy: Primary Aged Children

The mean scores for the actigraphy variables for the primary aged children on both weekdays and weekends are shown in Table 2. One control child failed to comply with wearing the actigraph at the weekend and so the control group’s actigraphy data were based on 14 participants.

Mixed-design ANOVAs with a within-subjects factor of “time” (weekday, weekend) and a between-subject factor of “group” (DCD, control) were conducted and the statistical test results are shown in Table 2. There were significant main effects for “time” for the variables *bedtime, sleep start time*, and *sleep duration* (i.e., children went to bed and to sleep later at the weekend than on weekdays and had shorter sleep durations at the weekend). There were no other significant main effects or interactions for actigraphy variables in this age group. There were trends for main effects although these did not meet the requirements for statistical significance following adjustment for FDR control. These were a main effect of “group” for the variable *wake up time* (qualified by a trend for an interaction with the DCD group waking up earlier at the weekend than did the control group) and also a trend for “group” for the variable *sleep duration* (for the DCD group to have shorter sleep durations).

### Actigraphy: Secondary Aged Children

The mean scores for the actigraphy variables for the secondary aged children on both weekdays and weekends separately are shown in Table 3. One control child failed to comply with wearing the actigraph at the weekend and so the control group’s actigraphy data were based on 14 participants.

Mixed-design ANOVAs with a within-subjects factor of “time” (weekday, weekend) and a between-subject factor of “group” (DCD, control) were conducted for all actigraphy variables. The ANOVA statistical test results are shown in Table 3. For the variable *sleep start time* there was a significant main effect for “time” (both the DCD and control groups had later sleep start time at the weekends compared to weekdays) but also a significant interaction (i.e., the difference between weekday and weekend *sleep start time* was greater for the control group than the DCD group because, compared to weekdays, the control group fell asleep significantly later at the weekend than the DCD group). For *wake up time*, there were also significant main effects for both “time” and “group” as well as a significant interaction (i.e., while both the DCD and control groups woke up earlier on weekdays than weekends, the DCD group generally woke earlier and the difference between weekdays and weekend wake up times was greater for the control group who woke up later than the DCD group at weekends).

There was a further main effect of “group” for *wake episodes* (i.e., the DCD group had more wake episodes) and main effects were seen of “time” for the variables *bedtime, sleep duration, wake minutes, wake after sleep onset, sleep efficiency*, and *activity mean* (i.e., compared to weekdays, weekend bedtime was later, total sleep duration was longer, there were more minutes of wake both during sleep and during the nighttime period (i.e., between going to bed and getting up) and sleep involved more movement). There were no other significant main effects or interactions for actigraphy variables in the secondary age group however again it is perhaps worth noting trends where the results were no longer statistically significant following adjustment for FDR control. These were non-significant trends of main effects for “group” in *fragmentation index* (with the DCD group having a higher fragmentation index) and also for “time” in relation to *activity SD* (greater night-to-night variability in movement scores at the weekend compared to weekdays).

### Children’s Sleep Habits Questionnaire

As presented in Table 4 the secondary school-aged adolescents with DCD had significantly higher CSHQ total scores than adolescents in the control group, indicating a greater severity of problems. There were no significant differences in the CSHQ total scores of primary aged children in the DCD and control groups. The number of children in each age/study group who scored above the clinical cut-off score of 41 appeared to be quite similar.

**Table 4:** **Median (interquartile range) scores for Children’s Sleep Habits Questionnaire variables for the primary and secondary school age children in the DCD and control groups**.

Scale (min–max)	DCD Group		Control Group		DCD vs. control split into primary and secondary
	Primary	Secondary	Primary	Secondary	
CSHQ total score (33–99)	45 (39–53)	46 (41–53)	43 (35–45)	41 (35–48)	Primary: *U* = 66.5; *p* = 0.056
					Secondary: *U* = 63.5; ***p* = 0.041**
Number of children scoring above the CSHQ total score cut-off (41)	10	12	9	9	–
CSHQ Bedtime resistance score (6–18)	6 (6–7)	6 (6–7)	6 (6–8)	6 (6–7)	Primary: *U* = 105.50; *p* = 0.742
					Secondary: *U* = 95.50; *p* = 0.392
CSHQ Sleep onset delay score (1–3)	2 (1–3)	2 (1–3)	1 (1–3)	2 (1–3)	Primary: *U* = 94.50; *p* = 0.419
					Secondary: *U* = 99.50; *p* = 0.569
CSHQ Sleep duration score (3–9)	4 (3–7)	5 (3–6)	3 (3)	3 (3–4)	Primary: *U* = 48.50; ***p* = 0.004**
					Secondary: *U* = 65.50; ***p* = 0.036**
CSHQ Sleep anxiety score (3–12)	5 (4–6)	5 (4–6)	4 (4–6)	4 (4–5)	Primary: *U* = 101.000; *p* = 0.612
					Secondary: *U* = 66.50; ***p* = 0.034**
CSHQ Night wakings score (3–9)	3 (3–4)	4 (3–4)	3 (3–4)	3 (3–4)	Primary: *U* = 106.50; *p* = 0.772
					Secondary: *U* = 88.50; *p* = 0.260
CSHQ Parasomnias score (3–21)	8 (8–11)	8 (7–10)	8 (7–8)	7 (7–9)	Primary: *U* = 77.50; *p* = 0.133
					Secondary: *U* = 75.00; *p* = 0.107
CSHQ Sleep-disordered breathing score (3–9)	3 (3–4)	3 (3–4)	3 (3–4)	3 (3)	Primary: *U* = 96.00; *p* = 0.410
					Secondary: *U* = 81.50; *p* = 0.065
CSHQ Daytime sleepiness score (3–24)	13 (10–19)	17 (12–20)	12 (8–13)	11 (10–18)	Primary: *U* = 73.00; *p* = 0.098
					Secondary: *U* = 67.50; *p* = 0.061

*CSHQ, Children’s Sleep Habits Questionnaire*.

*p-values <0.05 are marked in bold for ease of reading*.

Table 4 also presents the median scores of the CSHQ subscales. Primary aged children with DCD were reported to have significantly more problems on the subscale “sleep duration” (e.g., sleeping too little, lack of consistency in sleep duration). No other subscales differed between the DCD and control primary aged children. For secondary aged children, those with DCD also were reported to have significantly more problems on the subscale “sleep duration” and in addition to have significantly more sleep-related anxiety (e.g. being afraid of sleeping in the dark or sleeping alone, have trouble sleeping away from home). No other subscales differed between the DCD and control secondary aged children.

### Restless Legs Syndrome

No children in either the DCD or control group met the criteria for possible, probable, or definite RLS.

### Daytime Sleepiness, Fatigue, and Pre-Sleep Arousal

The mean scores and test statistics for the child report measures are shown in Table 5. For primary aged children, while daytime sleepiness did not differ between the groups, three scales of the PedsQL Multidimensional Fatigue Scale were significantly different between the groups; sleep/rest fatigue, cognitive fatigue and overall fatigue were all more problematic for the children with DCD than the controls. The subscale “general fatigue,” which includes more general/physical tiredness did not differ significantly between the groups. The primary aged DCD group also reported significantly more cognitive pre-sleep arousal; somatic pre-sleep arousal did not differ between the two groups.

**Table 5:** **Pediatric Daytime Sleepiness Scale, PedsQL Multidimensional Fatigue Scale, Pre-Sleep Arousal Scale, mean scores (and SDs) for the primary and secondary aged DCD and control groups**.

Scale (min–max)	DCD Group		Control Group		DCD vs. Control group comparison
	Primary	Secondary	Primary	Secondary	
PDSS (0–32)	13.20 (6.81)	17.33 (4.86)	11.60 (5.12)	12.47 (5.72)	Primary *t* = −0.727; *p* = 0.473
					Secondary *t* = −2.511; ***p* = 0.018**
Peds QL General Fatigue (0–100)	72.22 (16.04)	71.95 (17.29)	78.33 (13.19)	76.67 (15.41)	Primary *t* = 1.139; *p* = 0.264
					Secondary *t* = 0.789; *p* = 0.437
Peds QL Sleep/Rest Fatigue (0–100)	60.83 (11.77)	54.72 (21.47)	73.61 (14.06)	70.08 (16.36)	Primary *t* = 2.680; ***p* = 0.012**
					Secondary *t* = 2.203; ***p* = 0.036**
Peds QL Cognitive Fatigue (0–100)	50.42 (22.98)	53.61 (22.04)	71.39 (19.02)	80.00 (12.22)	Primary *t* = 2.722; ***p* = 0.011**
					Secondary *t* = 4.056; ***p* = 0.001**
Peds QL Total Fatigue (0–100)	61.76 (13.47)	60.09 (16.04)	74.07 (11.27)	75.65 (13.04)	Primary *t* = 2.716; ***p* = 0.011**
					Secondary *t* = 2.915; ***p* = 0.007**
PSAS – cognitive (8–40)	20.20 (6.20)	19.33 (5.6)	13.87 (4.52)	16.40 (4.67)	Primary *t* = −3.197; ***p* = 0.003**
					Secondary *t* = −1.557; *p* = 0.131
PSAS – somatic (8–40)	13.27 (4.33)	12.13 (3.11)	10.73 (2.96)	11.20 (3.08)	Primary *t* = 1.869; *p* = 0.072
					Secondary *t* = 0.826; *p* = 0.416

*PDSS, Pediatric Daytime Sleepiness Scale*.

*PSAS, Pre-Sleep Arousal Scale*.

*p-values <0.05 are marked in bold for ease of reading*.

For secondary aged children, daytime sleepiness was reported to be significantly worse for children with DCD than controls. As with the primary age group, the same three scales of the PedsQL Multidimensional Fatigue Scale were significantly different between the groups; sleep/rest fatigue, cognitive fatigue and overall fatigue were all more problematic for the children with DCD than the controls. No group differences in cognitive nor somatic pre-sleep arousal were found.

### Maternal Cognitions about Infant Sleep Questionnaire

There were no significant differences between the DCD and control groups’ mothers in terms of their thoughts about child sleep, for either age group. For the primary aged group the total MCISQ score for the mothers of children with DCD was 21.33 (SD = 11.21) and for the controls was 21.50 (SD = 10.32) (*U* = 99.5, *p* = 0.589). For the secondary aged group the total score for the mothers of children with DCD was 18.33 (SD = 12.38) and for the controls was 18.53 (SD = 6.91) (*t* = 0.055, *p* = 0.957). There were no significant group differences on any of the MCISQ subscale scores.

### Relationships between Severity of Motor Impairment and Sleep Measures for the DCD Group

Within the DCD group, there were no significant differences between any of the actigraphically defined variables, nor the CSHQ Total scores, for those children scoring above (*n* = 8) and below (*n* = 22) the 5th percentile on the MABC-2 (data available from the authors on request).

### Relationships between Daytime Sleepiness, Fatigue, Pre-Sleep Arousal, and Objective Sleep

The relationship between selected measures of daytime sleepiness (PDSS score), daytime fatigue (PedsQL General Fatigue and PedsQL Cognitive Fatigue), pre-sleep arousal (PSAS cognitive and PSAS somatic arousal) and key actigraphic objective sleep indices of sleep quality and quantity (WASO, *sleep efficiency, sleep latency, sleep duration*, and *activity during sleep*) were explored in the DCD and control groups separately. Correlation coefficients for the control and DCD groups can be seen in Table 6 where shaded cells indicate significant relationships for ease of comparison across the groups.

**Table 6:** **Correlations between measures of sleepiness, fatigue, pre-sleep arousal, and actigraphy in control children (top right, *n* = 30) and children with DCD (bottom left, *n* = 30)**.

	PDSS	PedsQL general	PedsQL cognitive	PSAS cognitive	PSAS somatic	WASO	Sleep efficiency	Sleep latency	Sleep duration	Activity
PDSS		*R* = −0.306	*R* = −0.463	*R* = 0.042	*R* = 0.281	*R* = −0.184	*R* = 0.126	*R* = −0.072	*R* = −0.007	*R* = −0.082
		*p* = 0.145	*p* = 0.023	*p* = 0.824	*p* = 0.132	*p* = 0.330	*p* = 0.507	*p* = 0.705	*p* = 0.972	*p* = 0.668

PedsQL general	*R* = −0.419		*R* = 0.718	*R* = −0.351	*R* = −0.623	*R* = −0.022	*R* = −0.032	*R* = 0.202	*R* = −0.097	*R* = 0.000
	*p* = 0.037		*p* < 0.001	*P* = 0.093	*p* = 0.001	*p* = 0.917	*p* = 0.882	*p* = 0.343	*p* = 0.652	*p* = 0.998

PedsQL cognitive	*R* = −0.397	*R* = 0.411		*R* = −0.237	*R* = −0.656	*R* = 0.252	*R* = −0.289	*R* = 0.370	*R* = −0.228	*R* = 0.150
	*p* = 0.030	*p* = 0.024		*p* = 0.265	*p* = 0.001	*p* = 0.234	*p* = 0.170	*p* = 0.075	*p* = 0.283	*p* = 0.484

PSAS cognitive	*R* = −0.403	*R* = −0.293	*r* = −0.287		*R* = 0.321	*R* = 0.165	*R* = −0.158	*R* = 0.012	*R* = −0.236	*R* = 0.295
	*p* = 0.046	*p* = 0.156	*p* = 0.163		*p* = 0.084	*p* = 0.384	*p* = 0.405	*p* = 0.950	*p* = 0.209	*p* = 0.113

PSAS somatic	*R* = 0.105	*R* = −0.341	*R* = −0.121	*R* = 0.557		*R* = −0.198	*R* = 0.192	*R* = −0.223	*R* = 0.054	*R* = −0.163
	*p* = 0.580	*p* = 0.095	*p* = 0.524	*p* = 0.001		*p* = 0.294	*p* = 0.310	*p* = 0.237	*p* = 0.778	*p* = 0.389

WASO	*R* = 0.419	*R* = −0.037	*R* = −0.629	*R* = 0.542	*R* = 0.330		*R* = −0.899	*R* = 0.293	*R* = −0.712	*R* = 0.736
	*p* = 0.021	*p* = 0.861	*p* = 0.001	*p* = 0.002	*p* = 0.075		*p* < 0.001	*p* = 0.117	*p* < 0.001	*p* < 0.001

Sleep efficiency	*R* = −0.452	*R* = 0.281	*R* = 0.652	*R* = −0.560	*R* = −0.227	*R* = −0.837		*R* = −0.614	*R* = 0.818	*R* = −0.785
	*p* = 0.012	*p* = 0.174	*p* < 0.001	*p* < 0.001	*p* = 0.228	*p* < 0.001		*p* < 0.001	*p* < 0.001	*p* < 0.0001

Sleep latency	*R* = 0.295	*R* = −0.412	*R* = −0.356	*R* = 0.211	*R* = −0.070	*R* = 0.082	*R* = −0.480		*R* = −0.420	*R* = 0.338
	*p* = 0.113	*p* = 0.041	*p* = 0.081	*p* = 0.263	*p* = 0.712	*p* = 0.666	*p* = 0.007		*p* = 0.021	*p* = 0.068

Sleep duration	*R* = −0.340	*R* = 0.103	*R* = 0.500	*R* = −0.441	*R* = −0.018	*R* = −0.538	*R* = 0.745	*R* = −0.221		*R* = −0.659
	*p* = 0.066	*p* = 0.623	*p* = 0.011	*p* = 0.015	*p* = 0.923	*p* = 0.002	*p* < 0.001	*p* = 0.240		*p* < 0.001

Activity	*R* = 0.426	*R* = −0.202	*R* = −0.535	*R* = 0.536	*R* = 0.248	*R* = 0.830	*R* = −0.886	*R* = 0.292	*R* = −0.615	
	*p* = 0.019	*p* = 0.333	*p* = 0.006	*p* = 0.002	*p* = 0.187	*p* < 0.001	*p* < 0.001	*P* = 0.118	*p* < 0.001	

*PDSS, Pediatric Daytime Sleepiness Scale*.

*PSAS, Pre-Sleep Arousal Scale*.

*WASO, Wake after sleep onset (actigraphy)*.

*Shaded cells indicate statistically significant correlations (*p* < 0.05)*.

In summary, there were differences and similarities in the pattern of relationships across the two groups. For the control children, daytime sleepiness was only correlated with cognitive fatigue (although the correlation is negative, low sleepiness scores indicated increased fatigue). By contrast, for children with DCD, increased daytime sleepiness was associated with more general fatigue, more cognitive pre-sleep arousal and a range of objective sleep variables indicating more disturbed sleep. General fatigue and cognitive fatigue were significantly positively correlated with each other for both groups. For the control group, both fatigue measures were correlated with somatic pre-sleep arousal (more fatigue associated with more pre-sleep somatic arousal). Such a relationship was not present for children with DCD and instead fatigue measures were variously associated with objective sleep measures; worse daytime fatigue was associated with more WASO, taking longer to fall asleep, more activity during sleep, lower sleep efficiency, and shorter sleep duration. Cognitive pre-sleep arousal was correlated with a range of objective sleep measures (increased cognitive arousal was associated with indicators of increased sleep disturbance) but this relationship was only seen in the DCD group. The relationships between some of the objective sleep measures were similar for both groups (apart from the negative correlation seen between sleep duration and sleep latency which was seen only in the control group).

## Discussion

This study is the first to report the objective sleep patterns of children with DCD and to explore these in relation to a range of other sleep and potentially relevant behavior measures gathered from the child and parent. The results suggest that, compared to controls, there are some differences in the sleep patterns of adolescents with DCD, both as reported by parents and also as assessed by actigraphy. Related behavioral measures of increased pre-sleep arousal, aspects of fatigue and daytime sleepiness also differed between the groups and were reported to be more severe by the children with DCD across the age range. The precise nature of the differences appeared to vary as a function of the child’s age. Some relationships between pre-sleep arousal, fatigue and daytime sleepiness, and the objective sleep variables were common to children with and without DCD although there were also patterns of associations which were specific to the DCD group.

Parents of both the primary and secondary school groups with DCD reported their children to have less sleep (and, increased sleep-related anxiety for the older age group) compared to controls. These parent reports show some congruence with the actigraph assessments; compared to controls differences in the timing of sleep were seen for secondary aged children with DCD, along with indicators of reduced sleep quality. Unsurprisingly, there were also differences in some actigraphy sleep variables between the weekdays and weekends that were seen in both children with and without DCD. Such differences were not the focus of the current study. However, of note was where the magnitude of the difference between weekdays and weekends varied between the control group and children with DCD given that weekday sleep patterns are perhaps more likely to be constrained by external factors (e.g., the need to get up for school) whereas weekend schedules could be considered to reflect more “natural” sleep patterns.

The objective sleep of primary aged children with DCD did not differ from controls, although there were non-significant trends to suggest they might have less sleep overall and tended to wake up somewhat earlier, particularly at weekends, compared to controls. Primary aged children with DCD reported more pre-sleep cognitive arousal than the control children. It is perhaps surprising that neither the parent report nor actigraphy suggests that there were any sleep onset difficulties since elevated pre-sleep cognitive arousal has previously shown to be associated with insomnia in children (47). A clearer understanding of the nature of any mental activity, and if it differs from the mental activity of children with insomnia, would be a helpful extension to this work.

The secondary age group of children with DCD showed a different pattern of sleep to their controls. Compared to the controls, the timing of their weekend sleep period appeared to be advanced (i.e., the time they fell asleep and woke up was earlier). While both the DCD and control groups went to sleep and woke up later at the weekend compared to weekdays, the magnitude of this difference was significantly greater for the control group. This is of note since adolescence is a time when a delay in the sleep phase can be expected so that there is a natural tendency and preference to go to sleep later and wake up later and to take longer to fall asleep even after extended periods awake due to both physiological (53) and psycho-social factors (54).

Such a weekend shift is generally considered to be undesirable and greater variability is associated with more daytime difficulties (55–57). Normally, consistency in weekday/weekend times is encouraged; but only when the regular sleep-wake times allow for the individual to obtain sufficient sleep. Results for the older group suggest that the quality of the sleep that they do get might not be good and perhaps, in such circumstances, catching up on sleep at the weekend might afford some benefits. It is also possible that it is the ability to cope with the consequences of sleep loss that distinguishes the children with DCD from the control group.

The reasons why the secondary aged DCD group do not show the same degree of “normal” shift needs to be explored. Their increased wakes from sleep could result in tiredness sufficient to override any natural tendency to delay their sleep phase. In support of this, adolescents with DCD reported more daytime sleepiness than the control group, which is of note given that sleepiness is common anyway in otherwise healthy adolescents (31). Reasons for the DCD adolescents’ increased wakings need to be explored by further investigations to determine the contribution of primary sleep disorders (e.g., a parasomnia or a sleep-related breathing disorder), any medical or psychological factors and environmental causes. The latter includes the possibility that the activities in which the children with DCD engage have a disruptive effect upon sleep or that engagement in specific activities have a particular impact on children with DCD. For example, the use of electronic media has been noted to be associated with sleep disruption in typically (58) and atypically developing (59) young people and group differences in the impact of such activities upon sleep have also been reported (60). It is not known whether use of electronic media is relevant for understanding the sleep of young people with DCD although one study (of 5- to 7-year-olds) reported that compared to a control group, children with DCD were more likely to report engaging in socially isolated, quieter, out-of-school activities, and also had a more limited range of participation experiences (61).

Interestingly, unlike the primary aged children with DCD, adolescents with DCD did not report increased pre-sleep arousal; however, their parents did consider these adolescents to have increased sleep anxiety (along with inadequate sleep duration). Such a lack of correspondence between parent and child informant highlights the usefulness of having multiple informants – and also the complexities in explaining any incongruous findings! Previous work has suggested that parents might under-report sleep difficulties when compared with child reports, not least because they might be unaware of more “subjectively” construed difficulties (62, 63). It may be that the adolescents with DCD are not reporting symptoms of pre-sleep arousal, either by choice or because they are unaware of it (at the time or in recollection). Alternatively, reporting bias may be relevant if parents of children with DCD are misconstruing their children’s pre-sleep state and behavior. Clarification of this is required since it has implications for potential target areas for intervention.

It should be noted that the nature of the parent-reported sleep problems differed in the current study compared to our earlier survey, where bedtime resistance, parasomnias, and daytime sleepiness were emphasized (30). The difference in the approach to the diagnosis of DCD in the samples of the two studies is likely to be relevant to making sense of this discrepancy. Furthermore, the variability of the types of parasomnias reported in the earlier study, in itself suggested that no particular parasomnia was a “characteristic” of the sleep of young people with DCD. Many parasomnias can, in susceptible individuals, of course be triggered by sleep deprivation or might be related to stress and so might be indicative of other underlying concerns (64).

In an attempt to better understand the nature of the sleep disturbances experienced by young people with DCD we included an assessment of RLS in the current study. This was considered important since this condition can be associated with sleep onset difficulties (and, therefore, sleep loss and daytime sleepiness) and, where enquired about, has been found to be more common in children (39, 65) than was previously thought to be the case. It is also commonly associated with PLMs during sleep (stereotypical limb jerks during sleep) which, because they can be related to arousals from sleep, may result in daytime tiredness. As these are movement-related sleep disorders, it was considered an omission that these had not been examined in children with some atypical aspects of their movement. However, results suggested that there were no indications, in any child, that RLS was present. This does not rule out the possibility that PLMs are present as these are only detected by use of PSG.

It is interesting that general physical fatigue was not reported to be a problem for children with DCD whereas sleep-related fatigue (e.g., sleepiness) and cognitive fatigue (e.g., problems with concentration and memory) were. There have been a couple of reports of ill-defined daytime “tiredness” in children with DCD [e.g., Ref. (30, 66)] and while the nature of this daytime impairment is not well-understood most common interpretations suggest that daytime fatigue is of a physical nature, arising because the children’s movement is less efficient/more effortful (67, 68). It is possible that this is an assumption. Definitions of fatigue are complex but the results of the current study suggest that attempts to isolate feelings of muscular fatigue from actual sleepiness are important as is determining whether daytime cognitive fatigue such as impaired memory and concentration arise as a result of sleep inadequacy, as has been documented in other groups of children (69–71). Clearer understanding of the children’s problems, and any underlying mechanisms, is significant for both assessment and treatment planning.

Teasing apart aspects of sleep, sleepiness, and fatigue is complicated by the inter-relations between these variables. In both TD controls and children with DCD, our exploratory correlative analyses showed associations between some measures of sleepiness and aspects of fatigue as well as relationships between these variables and between many of the actigraph variables, as might be expected. However, of particular interest was that relationships between objective sleep variables and selected measures of daytime functioning (daytime sleepiness, general fatigue, cognitive fatigue, and cognitive pre-sleep arousal) were only seen in the children with DCD and not in the control children. While causal relationships have yet to be established, these preliminary results emphasize the importance of considering sleep as salient in understanding some of the daytime difficulties of children with DCD. It was also of interest that cognitive pre-sleep arousal was not correlated with sleep latency but was associated with other objective sleep parameters reflecting quantity of sleep and quality of sleep *after* sleep onset. Such results are in line with neurocognitive explanations of insomnia, which suggest that elevated cognitive activity associated with insomnia (in adult samples) may not be causal of sleep difficulties but rather reflect differences in sleep associated brain activity (72). Polysomnographic studies of sleep in children with DCD would further understanding about whether atypical sleep microstructure was present in this group and also allow exploration of the link between polysomnographic aspects of sleep and motor function. For example, in TD children, motor skill performance on a finger tapping sequence task was better for children who had fewer slow spindles, more fast spindles, and faster slow waves during sleep (73). Of note, in the same study, was that overnight sleep-related improvements in accuracy were greatest for the children with a high density of slow spindles and slower slow waves (i.e., those features associated with poorer initial performance).

How any non-motor difficulties (of sleep or daytime functioning) are related to the motor difficulties in DCD remains to be delineated. Cairney and colleagues (16) adopt a useful conceptual framework to consider possible causal pathways between exposure to stress and psychological distress using Pearlin’s stress process model (17, 18). Cairney and colleagues explain that although DCD might not be a cause of stress *per se*, it can give rise to stressful experiences for children at home, at school and at play. The way in which these stressors interact with both the child’s personal resources and their networks of social support will determine psychological and social outcomes for the child. Importantly, the reciprocal relationships between sleep and a child’s emotional and behavioral functioning have been described (19, 25, 74), highlighting intervention possibilities (for sleep or daytime stressors) which could reduce any bi-directional associations.

However, one must interpret the current results in the light of the study’s limitations. While multiple comparisons were controlled for in our primary analyses of the actigraphy variables, such rigor was not applied to our more exploratory investigations; these results must be interpreted with appropriate caution. The sample sizes were small, and in line with the fact that DCD is more common in boys (1), girls were under-represented. Future work with larger samples is required to both confirm the results and enable investigation of any gender differences and also the impact of co-occurring conditions such as ADHD or ASD. While the inclusion of children with comorbidities helps ensure that the sample is representative of children with DCD and, in the current study, their exclusion did not change the outcome of the analyses, these co-occurring conditions may be important for accurate and refined interpretation of the findings.

Matters related to some of the assessment tools should also be considered. RLS was investigated using a novel tool and the validation of this is still underway. However this tool was devised by an international group of key clinicians and researchers in the field, with items based upon the most recent research findings and diagnostic criteria. It is also phrased in such a way to be accessible to children is intended to be more appropriate for use with children than other existing tools (75). A further limitation is that actigraphy estimates sleep/wake states on the basis of movement patterns. Although the scoring algorithms are validated with child and adult populations, the validity of this tool for assessing sleep in children with DCD remains to be determined. In addition, some aspects of sleep cannot be assessed by actigraphy and future studies using PSG to document sleep architecture (and potentially identify some sleep disorders which can only be diagnosed on the basis of PSG) would be helpful. Equally interesting would be qualitative studies with children and parents and the use of sleep/daily diaries to explore behavioral and environmental factors which might be related to the weekend timing of sleep patterns in young people with DCD.

## Conclusion

In conclusion, based on our analysis of two small samples of primary and secondary school-aged young people with DCD compared to controls, there were some differences in the sleep patterns of adolescents with DCD. There were also differences, for both age groups, in related behavioral measures of pre-sleep arousal, aspects of fatigue, and daytime sleepiness. Group differences and the patterns of relationships between variables appeared to vary as a function of the young person’s age. A clearer understanding of the nature and development of the sleep disturbance and the relationship between sleep and daytime functioning in young people with DCD is of importance for future research. It also has implications for assessment and management at an individual clinical level. These preliminary results support the need for a review of sleep (including separate attention to weekday and weekend sleep patterns) to form part of routine screening of young people with DCD, particularly during adolescence.

## Author Contributions

LW, Reader in Psychology Oxford Brookes University BSc DPhil CPsychol. MS, Research Assistant Oxford Brookes University BSc, Registered Nurse. AB, Professor in Psychology Oxford Brookes University BA, PhD, CPsychol, AFBPsS. The study was conceived by LW and AB. All authors (LW, MS, and AB) made a substantial contribution to the design, data acquisition, analysis, and interpretation and were involved in drafting and finalizing the paper. All authors agree to be accountable for all aspects of the work.

## Conflict of Interest Statement

The authors declare that the research was conducted in the absence of any commercial or financial relationships that could be construed as a potential conflict of interest.

## References

[1] American Psychiatric Association. Diagnostic and Statistical Manual of Mental Disorders: DSM-5. Washington, DC: American Psychiatric Association (2013).

[2] Cantell M Kooistra L Chapter 2, Long-term outcomes of developmental coordination disorder. In: Cermak SA Larkin D, editors. Developmental Coordination Disorder. Albany: Thomson Learning (2002). p. 23–38.

[3] Blank R Smits-Engelsman B Polatajko H Wilson P European Academy of Childhood Disability (EACD): recommendations on the definition, diagnosis and intervention of developmental coordination disorder (long version). Dev Med Child Neurol (2012) 54:54–93. 10.1111/j.1469-8749.2011.04175.x 22171930

[4] American Psychiatric Association. DSM-IV-TR. Diagnostic and Statistical Manual of Mental Disorders. 4th ed Washington, DC: American Psychiatric Association (2000).

[5] Lingam R Hunt L Golding J Jongmans M Emond A Prevalence of developmental coordination disorder using the DSM-IV at 7 years of age: a UK population based study. Pediatrics (2009) 123:e693–700. 10.1542/peds.2008-1770 19336359

[6] Barnett AL Is there a ‘movement thermometer’ for developmental coordination disorder? Curr Dev Disord Rep (2014) 1:132–9. 10.1007/s40474-014-0011-9

[7] Wilson PH Ruddock S Smits-Engelsman B Polatajko H Blank R. Understanding performance deficits in developmental coordination disorder: a meta-analysis of recent research. Dev Med Child Neurol (2013) 55(3):217–28. 10.1111/j.1469-8749.2012.04436.x 23106668

[8] Hands B Larkin D Physical fitness differences in children with and without motor learning difficulties. Eur J Spec Needs Educ (2006) 21:447–56. 10.1080/08856250600956410

[9] Cairney J Veldhuizen S Szatmari P. Motor coordination and emotional-behavioral problems in children. Curr Opin Psychiatry (2010) 23(4):324–9. 10.1097/YCO.0b013e32833aa0aa 20520549

[10] Cairney J, editor. Developmental Coordination Disoder and Its Consequences. Toronto: University of Toronto Press (2015).

[11] Poulsen AA Johnson H Ziviani JM. Participation, self-concept and motor performance of boys with developmental coordination disorder: a classification and regression tree analysis approach. Aust Occup Ther J (2011) 58:95–102. 10.1111/j.1440-1630.2010.00880.x 21418232

[12] Piek JP Barrett NC Allen LSR Jones A Louise M. The relationship between bullying and self-worth in children with movement coordination problems. Br J Educ Psychol (2005) 75:453–63. 10.1348/000709904X24573 16238876

[13] Pratt ML Hill E Anxiety profiles in children with and without developmental coordination disorder. Res Dev Disabil (2011) 32:1253–9. 10.1016/j.ridd.2011.02.006 21377831

[14] Lingam R Jongmans MJ Ellis M Hunt LP Golding J Emond A. Mental health difficulties in children with developmental coordination disorder. Pediatrics (2012) 129:E882–91. 10.1542/peds.2011-1556 22451706

[15] Kirby A Williams N Thomas M Hill E. Self-reported mood, general health, wellbeing and employment status in adults with suspected DCD. Res Dev Disabil (2013) 34:1357–64. 10.1016/j.ridd.2013.01.003 23417140

[16] Cairney J Rigoli D Piek J Developmental coordination disorder and internalizing problems in children: the environmental stress hypothesis elaborated. Dev Rev (2013) 33(3):224–38. 10.1016/j.dr.2013.07.002

[17] Pearlin LI The sociological study of stress. J Health Soc Behav (1989) 30(3):241–56. 10.2307/2136956 2674272

[18] Pearlin LI Lieberman MA Menaghan EG Mullan JT The stress process. J Health Soc Behav (1981) 22(4):337–56. 10.2307/2136676 7320473

[19] Gregory AM Sadeh A Annual research review: sleep problems in childhood psychiatric disorders – a review of the latest science. J Child Psychol Psychiatry (2016) 57(3):296–317. 10.1111/jcpp.12469 26412255

[20] Astill RG Van der Heijden KB Van IJzendoorn MH Van Someren EJW. Sleep, cognition, and behavioral problems in school-age children: a century of research meta-analyzed. Psychol Bull (2012) 138(6):1109–38. 10.1037/a0028204 22545685

[21] Cohen S Conduit R Lockley SW Rajaratnam SM Cornish KM. The relationship between sleep and behavior in autism spectrum disorder (ASD): a review. J Neurodev Disord (2014) 6(1):44. 10.1186/1866-1955-6-44 25530819 PMC4271434

[22] Greene G Gregory AM Fone D White J. Childhood sleeping difficulties and depression in adulthood: the 1970 British cohort study. J Sleep Res (2015) 24:19–23. 10.1111/jsr.12200 25178397

[23] Hakim F Kheirandish-Gozal L Gozal D. Obesity and altered sleep: a pathway to metabolic derangements in children? Semin Pediatr Neurol (2015) 22(2):77–85. 10.1016/j.spen.2015.04.006 26072337 PMC4466552

[24] Roberts RE Duong HT. Depression and insomnia among adolescents: a prospective perspective. J Affect Disord (2013) 148(1):66–71. 10.1016/j.jad.2012.11.049 23261135 PMC3644326

[25] Richdale AL Schreck KA Sleep problems in autism spectrum disorders: prevalence, nature, & possible biopsychosocial aetiologies. Sleep Med Rev (2009) 13(6):403–11. 10.1016/j.smrv.2009.02.003 19398354

[26] Tietze AL Blankenburg M Hechler T Michel E Koh M Schlüter B Sleep disturbances in children with multiple disabilities. Sleep Med Rev (2012) 16(2):117–27. 10.1016/j.smrv.2011.03.006 21620745

[27] Ren J Guo W Yan JH Liu G Jia F Practice and nap schedules modulate children’s motor learning. Dev Psychobiol (2016) 58(1):107–19. 10.1002/dev.21380 26582507

[28] van Schalkwijk FJ Benjamins JS Migliorati F de Nooijer JA van Someren EJ van Gog T The role of sleep timing in children’s observational learning. Neurobiol Learn Mem (2015) 125:98–105. 10.1016/j.nlm.2015.08.003 26303022

[29] Scabar A Devescovi R Blason L Carrozzi M. Comorbidity of DCD and SLI: significance of epileptiform activity during sleep. Child Care Health Dev (2006) 32(6):733–9. 10.1111/j.1365-2214.2006.00705.x 17018048

[30] Barnett AL Wiggs L. Sleep behaviour in children with developmental co-ordination disorder. Child Care Health Dev (2012) 38(3):403–11. 10.1111/j.1365-2214.2011.01260.x 21668466

[31] Wolfson AM O’Malley EB Sleep-related problems in adolescence and emerging adulthood. In: Morin CM Espie CA, editors. The Oxford Handbook of Sleep and Sleep Disorders. Oxford: Oxford University Press (2012). p. 746–68.

[32] Henderson SE Sugden DA Barnett A Movement Assessment Battery for Children – 2^nd^ Edition. London: Pearson (2007).

[33] Dunn LM Dunn DM Sewell J Styles B Brzyska N Shamsan Y The British Picture Vocabulary Scale, Third Edition. (BPVS-3). London: GL Assessment (2009).

[34] Glenn S Cunningham C. Performance of young people with Down syndrome on the Leiter-R and British picture vocabulary scales. J Intellect Disabil Res (2005) 49(4):239–44. 10.1111/j.1365-2788.2005.00643.x 15816810

[35] Goodman R. The strengths and difficulties questionnaire: a research note. J Child Psychol Psychiatry (1997) 38:581–6. 10.1111/j.1469-7610.1997.tb01545.x 9255702

[36] Sadeh A. The role and validity of actigraphy in sleep medicine: an update. Sleep Med Rev (2011) 15(4):259–67. 10.1016/j.smrv.2010.10.001 21237680

[37] Gruber R Sadeh A Raviv A. Instability of sleep patterns in children with attention-deficit/hyperactivity disorder. J Am Acad Child Adolesc Psychiatry (2000) 39(4):495–501. 10.1097/00004583-200004000-00019 10761352

[38] Owens JA Spirito A McGuinn M The Children’s Sleep Habits Questionnaire (CSHQ): psychometric properties of a survey instrument for school-aged children. Sleep (2000) 23(8):1043–51. 10.1037/t33022-000 11145319

[39] Picchietti DL Bruni O de Weerd A Durmer JS Kotagal S Owens JA Pediatric restless legs syndrome diagnostic criteria: an update by the International Restless Legs Syndrome Study Group. Sleep Med (2013) 14(12):1253–9. 10.1016/j.sleep.2013.08.778 24184054

[40] Allen RP Picchietti D Hening WA Trenkwalder C Walters AS Montplaisi J Restless legs syndrome: diagnostic criteria, special considerations, and epidemiology. A report from the restless legs syndrome diagnosis and epidemiology workshop at the National Institutes of Health. Sleep Med (2003) 4(2):101–19. 10.1016/S1389-9457(03)00010-8 14592341

[41] Picchietti DL Arbuckle RA Abetz L Durmer JS Ivanenko A Owens JA Pediatric restless legs syndrome: analysis of symptom descriptions and drawings. J Child Neurol (2011) 26(11):1365–76. 10.1177/0883073811405852 21636777

[42] Drake C Nickel C Burduvali E Roth T Jefferson C Pietro B. The pediatric daytime sleepiness scale (PDSS): sleep habits and school outcomes in middle-school children. Sleep (2003) 26(4):455–8. 10.1037/t02761-000 12841372

[43] Lewandowski AS Toliver-Sokol M Palermo TM. Evidence-based review of subjective pediatric sleep measures. J Pediatr Psychol (2011) 36(7):780–93. 10.1093/jpepsy/jsq119 21227912 PMC3146754

[44] Varni JW Burwinkle T Katz ER Meeske K Dickinson P The PedsQL^™^ in pediatric cancer: reliability and validity of the pediatric quality of life inventory^™^ generic core scales, multidimensional fatigue scale, and cancer module. Cancer (2002) 94:2090–106. 10.1002/cncr.10428 11932914

[45] Varni JW Burwinkle TM Szer IS. The PedsQL Multidimensional Fatigue Scale in pediatric rheumatology: reliability and validity. J Rheumatol (2004) 31(12):2494–500. 15570657

[46] Nicassio PM Mendlowitz DR Fussell JJ Petras L The phenomenology of the pre-sleep state – the development of the pre-sleep arousal scale. Behav Res Ther (1985) 23:263–71. 10.1016/0005-7967(85)90004-X 4004706

[47] Gregory AM Willis TA Wiggs L Harvey AG STEPS Team. Presleep arousal and sleep disturbances in children. Sleep (2008) 31(12):1745–7. 19090331 10.1093/sleep/31.12.1745 PMC2603482

[48] Morrell JM. The role of maternal cognitions in infant sleep problems as assessed by a new instrument, the maternal cognitions about infant sleep questionnaire. J Child Psychol Psychiatry (1999) 40(2):247–58. 10.1111/1469-7610.00438 10188707

[49] Williams JA Zimmerman FJ Bell JF. Norms and trends of sleep time among US children and adolescents. JAMA Pediatr (2013) 167(1):55–60. 10.1001/jamapediatrics.2013.423 23403646 PMC5814130

[50] Ohayon MM Carskadon MA Guilleminault C Vitiello MV. Meta-analysis of quantitative sleep parameters from childhood to old age in healthy individuals: developing normative sleep values across the human lifespan. Sleep (2004) 27(7):1255–73. 15586779 10.1093/sleep/27.7.1255

[51] Benjamini Y Hochberg Y Controlling the false discovery rate: a practical and powerful approach to multiple testing. J R Stat Soc Series B (1995) 57:289–300.

[52] Glickman ME Rao SR Schultz MR False discovery rate control is a recommended alternative to Bonferroni-type adjustments in health studies. J Clin Epidemiol (2014) 67:850–7. 10.1016/j.jclinepi.2014.03.012 24831050

[53] Taylor DJ Jenni OG Acebo C Carskadon MA. Sleep tendency during extended wakefulness: insights into adolescent sleep regulation and behavior. J Sleep Res (2005) 14(3):239–44. 10.1111/j.1365-2869.2005.00467.x 16120098

[54] Malone SK. Early to bed, early to rise?: an exploration of adolescent sleep hygiene practices. J Sch Nurs (2011) 27(5):348–54. 10.1177/1059840511410434 21606219

[55] Lee YJ Park J Kim S Cho SJ Kim SJ. Academic performance among adolescents with behaviorally induced insufficient sleep syndrome. J Clin Sleep Med (2015) 11(1):61–8. 10.5664/jcsm.4368 25515277 PMC4265661

[56] Perez-Lloret S Videla AJ Richaudeau A Vigo D Rossi M Cardinali DP A multi-step pathway connecting short sleep duration to daytime somnolence, reduced attention, and poor academic performance: an exploratory cross-sectional study in teenagers. J Clin Sleep Med (2013) 9(5):469–73. 10.5664/jcsm.2668 23674938 PMC3629321

[57] Wong ML Lau EY Wan JH Cheung SF Hui CH Mok DS. The interplay between sleep and mood in predicting academic functioning, physical health and psychological health: a longitudinal study. J Psychosom Res (2013) 74(4):271–7. 10.1016/j.jpsychores.2012.08.014 23497826

[58] Cain N Gradisar M. Electronic media use and sleep in school-aged children and adolescents: a review. Sleep Med (2010) 11(8):735–42. 10.1016/j.sleep.2010.02.006 20673649

[59] Mazurek MO Shattuck PT Wagner M Cooper BP Prevalence and correlates of screen-based media use among youths with autism spectrum disorders. J Autism Dev Disord (2012) 42(8):1757–67. 10.1007/s10803-011-1413-8 22160370 PMC3536490

[60] Engelhardt CR Mazurek MO Sohl K. Media use and sleep among boys with autism spectrum disorder, ADHD, or typical development. Pediatrics (2013) 132:1081–9. 10.1542/peds.2013-2066 24249825

[61] Jarus T Lourie-Gelberg Y Engel-Yeger B Bart O. Participation patterns of school-aged children with and without DCD. Res Dev Disabil (2011) 32(4):1323–31. 10.1016/j.ridd.2011.01.033 21324639

[62] Paavonen EJ Aronen ET Moilanen I Piha J Räsänen E Tamminen T Sleep problems of school-aged children: a complementary view. Acta Paediatr (2000) 89(2):223–8. 10.1111/j.1651-2227.2000.tb01220.x 10709895

[63] Meltzer LJ Avis KT Biggs S Reynolds AC Crabtree VM Bevans KB. The children’s report of sleep patterns (CRSP): a self-report measure of sleep for school-aged children. J Clin Sleep Med (2013) 9(3):235–45. 10.5664/jcsm.2486 23493949 PMC3578690

[64] American Academy of Sleep Medicine. International Classification of Sleep Disorders: Diagnostic and Coding Manual. 3rd ed Darien, IL: American Academy of Sleep Medicine (2014).

[65] Silvestri R Gagliano A Aricò I Calarese T Cedro C Bruni O Sleep disorders in children with attention deficit/hyperactivity disorder (ADHD) recorded overnight by video-polysomnography. Sleep Med (2009) 10(10):1132–8. 10.1016/j.sleep.2009.04.003 19527942

[66] Barnett AL Dawes H Wilmut K. Constraints and facilitators to participation in physical activity in teenagers with developmental co-ordination disorder: an exploratory interview study. Child Care Health Dev (2013) 39(3):393–403. 10.1111/j.1365-2214.2012.01376.x 22515369

[67] Chia LC Licari MK Guelfi KJ Reid SL. A comparison of running kinematics and kinetics in children with and without developmental coordination disorder. Gait Posture (2013) 38(2):264–9. 10.1016/j.gaitpost.2012.11.028 23266248

[68] Rivilis I Hay J Cairney J Klentrou P Liu J Faught BE. Physical activity and fitness in children with developmental coordination disorder: a systematic review. Res Dev Disabil (2011) 32:894–910. 10.1016/j.ridd.2011.01.017 21310588

[69] Fallone G Acebo C Arnedt JT Seifer R Carskadon MA. Effects of acute sleep restriction on behavior, sustained attention, and response inhibition in children. Percept Mot Skills (2001) 93(1):213–29. 10.2466/pms.2001.93.1.213 11693688

[70] Sadeh A Gruber R Raviv A. The effects of sleep restriction and extension on school-age children: what a difference an hour makes. Child Dev (2003) 74(2):444–55. 10.1111/1467-8624.7402008 12705565

[71] Vriend JL Davidson FD Corkum PV Rusak B Chambers CT McLaughlin EN. Manipulating sleep duration alters emotional functioning and cognitive performance in children. J Pediatr Psychol (2013) 38(10):1058–69. 10.1093/jpepsy/jst033 23720415

[72] Spiegelhalder K Regen W Baglioni C Nissen C Riemann D Kyle SD. Neuroimaging insights into insomnia. Curr Neurol Neurosci Rep (2015) 15(3):9. 10.1007/s11910-015-0527-3 25687698

[73] Astill RG Piantoni G Raymann RJ Vis JC Coppens JE Walker MP Sleep spindle and slow wave frequency reflect motor skill performance in primary school-age children. Front Hum Neurosci (2014) 8:910. 10.3389/fnhum.2014.00910 25426055 PMC4227520

[74] Gregory AM Sadeh A. Sleep, emotional and behavioral difficulties in children and adolescents. Sleep Med Rev (2012) 16(2):129–36. 10.1016/j.smrv.2011.03.007 21676633

[75] Walters AS Frauscher B Allen R Benes H Chaudhuri KR Garcia-Borreguero D Review of diagnostic instruments for the restless legs syndrome/Willis-Ekbom disease (RLS/WED): critique and recommendations. J Clin Sleep Med (2014) 10(12):1343–9. 10.5664/jcsm.4298 25348242 PMC4237530

